# Fungal Infections Identified with Multiplex PCR in Severe COVID-19 Patients during Six Pandemic Waves

**DOI:** 10.3390/medicina59071253

**Published:** 2023-07-05

**Authors:** Iulia Bogdan, Akash Reddy Reddyreddy, Aditya Nelluri, Ram Kiran Maganti, Felix Bratosin, Roxana Manuela Fericean, Catalin Dumitru, Paula Irina Barata, Gianina Tapalaga, Iosif Marincu

**Affiliations:** 1 Department XIII, Discipline of Infectious Diseases, “Victor Babes” University of Medicine and Pharmacy, 300041 Timisoara, Romania; iulia.georgianabogdan@gmail.com (I.B.); felix.bratosin@umft.ro (F.B.); imarincu@umft.ro (I.M.); 2 Doctoral School, “Victor Babes” University of Medicine and Pharmacy, 300041 Timisoara, Romania; manuela.fericean@umft.ro; 3 School of General Medicine, Bhaskar Medical College, Hyderabad 500075, India; drakashreddy28@gmail.com; 4 School of General Medicine, Sri Siddhartha Medical College, Tumakuru 572107, India; nelluriaditya@gmail.com; 5 School of General Medicine, Sri Devaraj Urs Academy of Higher Education and Research, Kolar 563101, India; ramkiran.maganti11@gmail.com; 6 Department of Obstetrics and Gynecology, “Victor Babes” University of Medicine and Pharmacy, 300041 Timisoara, Romania; dumitru.catalin@umft.ro; 7 Department of Physiology, Faculty of Medicine, “Vasile Goldis” Western University of Arad, 310025 Arad, Romania; 8 Center for Research and Innovation in Precision Medicine of Respiratory Diseases, “Victor Babes” University of Medicine and Pharmacy, 300041 Timisoara, Romania; 9 Department of Odontotherapy and Endodontics, Faculty of Dental Medicine, “Victor Babes” University of Medicine and Pharmacy, 300041 Timisoara, Romania; tapalaga.gianina@umft.ro

**Keywords:** COVID-19, SARS-CoV-2, fungal infections, multiplex PCR

## Abstract

*Background and Objectives*: With an increasing number of severe COVID-19 cases presenting with secondary fungal infections, this study aimed to determine the prevalence of fungal co-infections in severe COVID-19 patients across the six waves, identify the most common fungal pathogens associated with severe COVID-19, and explore any potential links between patient characteristics, therapeutic strategies, and the prevalence and type of fungal infection. *Materials and Methods*: A retrospective analysis was conducted on severe COVID-19 patients admitted to the Infectious Diseases and Pulmonology Hospital, “Victor Babes”, Romania, between March 2020 and August 2022. Samples were collected from respiratory specimens, blood, and urine, after which a standard nucleic acid extraction protocol was employed. Patients were divided into groups with and without fungal infections, identified using multiplex PCR. The groups were compared based on demographic data, comorbidities, pandemic wave number, and clinical outcomes. *Results*: Out of 288 patients, 96 (33.3%) had fungal infections, with *Candida* spp. being the most common. Patients with fungal infections had higher rates of obesity (35.4% vs. 21.4%, *p* = 0.010) and a higher Charlson comorbidity index (CCI > 2) (37.5% vs 25.0%, *p* = 0.027). Ventilator use was significantly higher in the fungal infection group (45.8% vs. 18.8%; *p* < 0.001), as was ICU admission (39.6% vs. 26.6%; *p* = 0.024) and mortality (32.3% vs 12.0%; *p* < 0.001). The distribution of different fungal species varied across the pandemic waves, with no statistical significance (*p* = 0.209). The mortality risk notably increased with the degree of drug resistance (OR for three or more drug resistances = 6.71, *p* < 0.001). The second, fourth, and fifth pandemic waves were significantly associated with higher mortality risk (OR = 3.72, 3.61, and 4.08, respectively, all *p* < 0.001). *Aspergillus* spp. and *Mucor* spp. infections were significantly associated with increased mortality risk (OR = 4.61 and 6.08, respectively, both *p* < 0.001). *Conclusions*: Our study indicates a significant presence of fungal co-infections among severe COVID-19 patients that is associated with increased morbidity and mortality, particularly in patients with drug-resistant infections. These findings underline the necessity for comprehensive diagnostic approaches and tailored treatment strategies in managing COVID-19 patients, especially during specific pandemic waves and in patients with particular fungal infections. Further research is required to understand the implications of these co-infections and their management.

## 1. Introduction

The global outbreak of the novel coronavirus disease 2019 (COVID-19), caused by the severe acute respiratory syndrome coronavirus 2 (SARS-CoV-2), has precipitated a significant global health crisis [1,2,3,4]. Whereas the primary focus has been on understanding and managing the viral pathogenesis and its complications [5,6,7,8], secondary complications, such as opportunistic fungal infections, have emerged as substantial contributors to patient morbidity and mortality [9,10,11]. However, the interplay between the virus and co-infections, particularly those of fungal nature, remains poorly understood, leaving a gap in our knowledge of disease progression and management [12].

The presence of fungal infections in critically ill patients, including those affected by COVID-19, is well documented [13,14,15]. Such secondary infections often lead to poorer prognosis, extended hospital stays, and increased healthcare costs [16,17]. Factors such as immunosuppression, mechanical ventilation, and the use of broad-spectrum antibiotics contribute to the high prevalence of these infections [18,19,20]. Nonetheless, the detection and identification of these infections pose a significant challenge due to the lack of rapid and sensitive diagnostic methods [21].

Conventional methods of detecting fungal infections, such as culture and microscopy, often yield slow and sometimes inaccurate results [22]. Additionally, serology-based tests may have issues with sensitivity and specificity [23]. Recent developments have seen the advent of newer technologies such as next-generation sequencing (NGS) and matrix-assisted laser desorption/ionization time-of-flight mass spectrometry (MALDI-TOF MS). However, these techniques require high technical expertise, expensive infrastructure, and often, more time than multiplex PCR for accurate results [24].

Multiplex polymerase chain reaction (PCR) has been acknowledged as a promising tool for the swift detection of fungal pathogens [25]. By enabling the simultaneous detection of multiple pathogens, multiplex PCR offers a potential solution to the issue of timely and accurate fungal identification [26,27]. Compared with other methods, multiplex PCR is more rapid, sensitive, and cost-effective and can provide a comprehensive view of the pathogens present in a sample [28]. However, despite its promise, the application of multiplex PCR in the context of severe COVID-19 patients has not been extensively explored [29,30].

Throughout the COVID-19 pandemic, the need to understand the epidemiology of fungal infections in severe COVID-19 patients became apparent [31]. Different waves of the pandemic offered a chance to observe patterns, prevalence, and changes in the fungal co-infections associated with COVID-19 [32,33,34]. Despite the wealth of information garnered from these waves, there is still a paucity of comprehensive studies focused on the fungal co-infections identified with multiplex PCR in severe COVID-19 patients across multiple pandemic waves [35].

This study, therefore, aimed to fill this research gap by undertaking a comprehensive analysis of the fungal infections identified using multiplex PCR in severe COVID-19 patients during the first six pandemic waves. The study hypothesized that there would be significant variability in the prevalence and type of fungal infections across the different waves, potentially influenced by changes in therapeutic strategies, virus variants, and patient demographics. The objectives of the study were to determine the prevalence of fungal co-infections in severe COVID-19 patients across the six waves, identify the most common fungal pathogens associated with severe COVID-19, and explore any potential links between patient characteristics, therapeutic strategies, and the prevalence and type of fungal infection.

## 2. Materials and Methods

### 2.1. Study Design and Ethics

This study was a retrospective analysis conducted between March 2020 and August 2022, during which six waves of the COVID-19 pandemic occurred. Patients included in the study were admitted to the Infectious Diseases and Pulmonology Hospital, “Victor Babes”, affiliated with the “Victor Babes” University of Medicine and Pharmacy from Timisoara, Romania. The study was designed to investigate the incidence and types of fungal infections in severe COVID-19 patients. Ethical approval for the study was obtained from the Institutional Review Board of the hospital. All patient data were anonymized before analysis to maintain confidentiality.

### 2.2. Patients’ Inclusion and Exclusion Criteria

Patients included in the study were adults (18 years or older) who had been diagnosed with severe COVID-19 as defined by the World Health Organization [36]. The diagnosis had been confirmed through a positive SARS-CoV-2 PCR test, and the paper records specified the ICD-10 diagnosis code for SARS-CoV-2 infection [37] and the patient’s consent to participate in medical research studies. The study also included a control group of patients with severe COVID-19 and a negative PCR and blood culture test at a 2:1 ratio. Patients were proportionally matched by age, gender, and vaccination status to reduce the bias for mortality risk assessment. Infections diagnosed within 48 h of hospital admission were classified as co-infections. Infections identified after 48 h of admission were classified as superinfections.

Patients were excluded from the study if they had a known history of immunodeficiency diseases, were receiving immunosuppressive therapy before the onset of COVID-19, or if they had a history of fungal infection before the COVID-19 diagnosis. Pregnant women, ICU-admitted patients, and patients who did not give consent for their medical data to be used for research purposes were also excluded. Additionally, patients with mild or moderate COVID-19 were excluded from the current study, as well as those identified with bacterial infections.

Exclusion from the study was also applied to patients identified with bacterial infections. These were detected via a combination of traditional blood culture methods and specific PCR assays. The specific PCR assays were employed to provide a rapid and accurate identification of the bacterial pathogens, whereas blood cultures were used as the gold-standard method for their detection. All the PCR assays for bacterial detection were performed as per previously published protocols [38]. Conventional blood cultures were performed as per standard guidelines outlined by the Clinical and Laboratory Standards Institute (CLSI) [39].

### 2.3. Study Materials and Variables

Clinical data were retrieved from the patients’ paper and electronic medical records. These data included demographic information, underlying comorbidities, laboratory test results, and treatments received. The primary study variable was the presence or absence of a fungal infection. Fungal infections were identified using multiplex PCR, a technique that allows for the simultaneous detection of multiple fungal pathogens in a single reaction. The primers used in the PCR reaction were targeted at specific sequences of the fungal pathogens of interest. Specifically, the primers for *Candida* spp., *Aspergillus* spp., *Mucor* spp., and *Rhizopus* spp. were used. These primers have been previously described and validated in the literature for their specificity and sensitivity in identifying the aforementioned fungal pathogens [40]. For this purpose, patient samples were obtained from respiratory specimens, blood, urine, and other relevant sites, depending on the clinical picture. The specific fungal pathogens detected by the multiplex PCR were also recorded.

Before the multiplex PCR procedure, the nucleic acids from the patient samples were extracted using a standard extraction protocol. For this process, we used the QIAamp DNA Mini Kit (QIAGEN, Hilden, Germany) following the manufacturer’s instructions. The extracted DNA was then quantified using a Qubit™ 4 Fluorometer (Thermo Fisher Scientific, Waltham, MA, USA) to ensure the suitability of the samples for the subsequent PCR analysis. The cDNA synthesis, crucial for the amplification of RNA viruses, was performed using the RevertAid First Strand cDNA Synthesis Kit (Thermo Fisher Scientific, Waltham, MA, USA) as per the manufacturer’s protocol. After this process, the DNA and cDNA samples were amplified using specific primers for the pathogens of interest during the multiplex PCR process. Venous blood was used as the sampling technique, and routine microbiological investigations were conducted at the medical microbiology laboratory using standard bacteriology guidelines. All isolates were first identified using the VITEK 2 GN and VITEK 2 GP ID cards (BioMérieux, Marcy-l’Etoile, France).

All the procedures involving the handling of patient samples, including the extraction of nucleic acids, the performance of PCR assays, and the culture of microorganisms, were conducted in a laboratory with biosafety level 2 (BSL-2) containment. This is in line with the recommended practices and procedures for working with agents that pose a moderate potential hazard to personnel and the environment [41].

The data collected from patients were classified based on the phase of the pandemic during which they were admitted to the hospital. The first wave of the pandemic in Romania was thought to have taken place from March to October 2020. During this period, the predominant variant in circulation was Wuhan-Hu-1 (NCBI Reference Sequence: NC 045512.2) [42]. Subsequently, the second wave of COVID-19 was observed from October 2020 to February 2021. This period was characterized by the prevalence of clade variants (S: D614G), which became the dominant viral strains [43]. Following this, the third wave of the pandemic swept across the nation from February to July 2021. The alpha variant (B.1.1.7) emerged as the primary circulating virus during this phase [44]. The fourth wave struck between July and December of 2021, during which the delta variant (B1617.2) of SARS-CoV-2 became the most prevalent strain [45,46]. The fifth wave, triggered by the omicron variant, occurred in Romania between December 2021 and March 2022 [47]. Lastly, the sixth wave stretched from March 2022 to July 2022, although the dominant strain during this phase was not specified in the original text [48]. It is important to note that each wave was defined by the dominant viral variant at the time, which could have implications on the severity of the disease, the transmissibility of the virus, and the effectiveness of the treatments used.

### 2.4. Statistical Analysis

Statistical analyses were performed using SPSS software version 26.0 (IBM, Chicago, IL, USA). Descriptive statistics were used to characterize the study population, presenting continuous variables as means and standard deviations and categorical variables as frequencies and percentages. The incidence of fungal infections was calculated as the number of new cases during the study period divided by the total number of severe COVID-19 patients. For the comparison of proportions, the χ^2^ and Fisher’s tests were employed, whereas for the comparison of group differences in nonparametric data, the Mann–Whitney test was used. Parametric continuous variables that followed a normal distribution were compared by mean and standard deviation using Student’s *t*-test (unpaired, independent samples). To assess the association between fungal infections and other variables (such as demographic characteristics, comorbidities, or treatments), logistic regression analyses were performed. Variables with a *p*-value of less than 0.05 in the univariate analysis were included in a multivariate logistic regression model. Odds ratios and 95% confidence intervals were calculated. All tests were two-sided, and *p*-values less than 0.05 were considered statistically significant.

## 3. Results

### 3.1. Patient Demographics

Among the patients examined, 96 had fungal infections, whereas 192 did not. The average age for those with fungal infections was slightly higher (64.6 ± 12.1 years) compared with those without infections (62.0 ± 11.5 years), although the difference was not statistically significant (*p* = 0.076). Sexual disparity was observed among the patients, with men constituting 63.5% of those with fungal infections and 55.7% of those without. However, this difference was not statistically significant (*p* = 0.205). A significant difference was found in the distribution of obesity, measured by body mass index (BMI > 29.9 kg/m^2^), with 35.4% of patients with fungal infections being obese compared with 21.4% of those without fungal infections (*p* = 0.010). The rate of COVID-19 vaccination (with ≥2 doses) was almost identical in both groups, at 9.4% for those with fungal infections and 10.4% for those without (*p* = 0.781). Smoking was slightly more prevalent among patients with fungal infections (43.8%) than those without (34.4%), but this difference was not statistically significant (*p* = 0.121).

Various pulmonary diseases were analyzed, including chronic bronchitis, chronic obstructive pulmonary disease (COPD), asthma, and pulmonary hypertension. Although higher prevalence rates were generally observed in the group with fungal infections, none of these differences reached statistical significance. A significant difference was observed in the Charlson comorbidity index (CCI > 2), with 37.5% of patients with fungal infections having a high CCI compared with 25.0% of those without infections (*p* = 0.027). The distribution of patients across the six pandemic waves was also analyzed, with no significant difference found (*p* = 0.272), as presented in Table 1.

### 3.2. Disease Management and Outcomes

Conventional blood cultures were performed more frequently in the group with fungal infections (64.6%) than in the group without fungal infections (56.3%), but this difference was not statistically significant (*p* = 0.175). Multiplex PCR tests were performed for all patients in both groups. The study also compared oxygen supplementation methods. Although AIRVO was employed slightly less in the group with fungal infections (68.8% vs. 73.4%), this difference was not statistically significant (*p* = 0.404). However, CPAP was used significantly less often in the group with fungal infections (36.5% vs. 51.0%; *p* = 0.019). Interestingly, ventilator use was significantly more frequent among patients with fungal infections (45.8% vs. 18.8%; *p* < 0.001), as described in Table 2.

**Figure 1:**
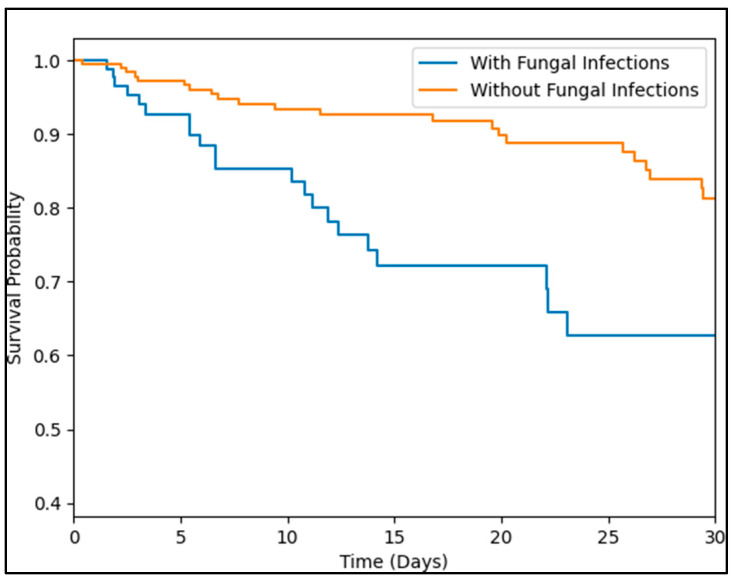
Kaplan–Meier analysis of survival between severe COVID-19 patients with and without fungal infections.

The timing of sample collection did not significantly differ between the two groups (*p* = 0.156). However, in terms of clinical outcomes, the study found significant differences. ICU admission was more common among patients with fungal infections (39.6% vs. 26.6%; *p* = 0.024), and these patients also spent more days in the ICU (12.8 ± 7.2 vs. 10.5 ± 6.9 days; *p* = 0.009). Furthermore, the time from symptom onset until death was slightly shorter in patients with fungal infections (13.6 ± 9.4 vs. 16.2 ± 8.0 days; *p* = 0.014), and mortality was significantly higher in this group (32.3% vs. 12.0%; *p* < 0.001). Lastly, the average hospital stay before discharge was longer for patients with fungal infections (18.8 ± 9.0 vs. 15.3 ± 9.7 days; *p* = 0.003). The Kaplan–Meier analysis of survival between severe COVID-19 patients with and without fungal infections identified a significantly higher probability of death among those with fungal infections (log-rank *p*-value = 0.003), as seen in Figure 1.

### 3.3. Identification of Fungal Species and Drug Resistance

The distribution of different fungal species varied across the pandemic waves, although the differences were not statistically significant (*p* = 0.209). *Candida* spp. was the most common fungal infection across all waves, with the highest prevalence in the first wave (71.4%) and the lowest in the third wave (35.0%). *Aspergillus* spp. also showed variability, with the highest prevalence in the third wave (55.0%) and the lowest in the second wave (30.8%). *Mucor* spp. was less commonly identified across all waves, with the highest detection rate in the first wave (20.0%) and then not being identified at all in the fourth and sixth waves. *Rhizopus* spp. was also less common and was not detected in the second wave; it reached its peak in the fourth wave (11.1%).

Clinical outcomes, including ICU admission and mortality, showed significant differences across the waves (*p* = 0.024 and *p* = 0.018, respectively). ICU admissions were the highest in the fourth wave (28.9%) and the lowest in the sixth wave (7.9%). Similarly, mortality was highest in the fourth wave (32.3%) and lowest in the sixth wave (3.2%), as described in Table 3.

For patients with *Candida* spp. infections, 19.6% had no drug resistance, whereas the majority displayed resistance to at least one drug (35.3% had resistance to one drug and 39.2% to two drugs). A small proportion of *Candida* spp. infections (5.9%) exhibited resistance to three or more drugs. *Aspergillus* spp. infections showed a similar pattern, with 19.4% having no drug resistance, 33.3% resistance to one drug, and 41.7% resistance to two drugs. A small percentage (5.6%) showed resistance to three or more drugs, as presented in Table 4.

Among the *Mucor* spp. infections, one-third showed no drug resistance, one-third showed resistance to one drug, and one-third showed resistance to two drugs. Notably, none of the *Mucor* spp. infections demonstrated resistance to three or more drugs. In the case of *Rhizopus* spp., no infections showed an absence of drug resistance. Half of these infections showed resistance to one drug, 16.7% to two drugs, and, notably, a substantial proportion (33.3%) showed resistance to three or more drugs.

### 3.4. Mortality Risk Assessment

Regarding drug resistance features, the mortality risk notably increased with the degree of drug resistance. Patients with no drug resistance had an odds ratio (OR) of 1.66 for mortality, although the result was not statistically significant (*p* = 0.062). However, a significant rise in mortality risk was observed for patients with one drug resistance (OR = 2.95, *p* = 0.001), two drug resistances (OR = 4.03, *p* < 0.001), and three or more drug resistances (OR = 6.71, *p* < 0.001). These findings indicate that drug resistance was a significant predictor of mortality among severe COVID-19 patients with fungal infections.

When the analysis was stratified by pandemic wave, the second, fourth, and fifth pandemic waves were significantly associated with higher mortality risk (OR = 3.72, 3.61, and 4.08, respectively, all *p* < 0.001). The third wave also showed a significant but lesser increase in mortality risk (OR = 1.40, *p* = 0.003). The first and sixth waves were not significantly associated with an increased risk of mortality.

**Figure 2:**
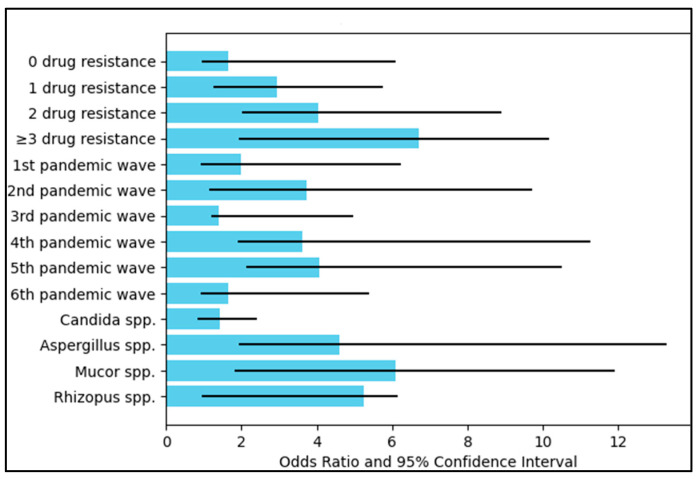
Risk factor analysis for mortality.

Concerning the type of fungal infection, *Aspergillus* spp. and *Mucor* spp. infections were significantly associated with increased mortality risk (OR = 4.61 and 6.08, respectively, both *p* < 0.001). Although *Rhizopus* spp. showed a high OR of 5.26, the result did not reach statistical significance (*p* = 0.107). *Candida* spp. infections did not show a significant increase in mortality risk (OR = 1.43, *p* = 0.194), as presented in Table 5 and Figure 2.

## 4. Discussion

### 4.1. Literature Findings

The current study provides valuable insights into the incidence, distribution, and clinical implications of fungal infections in severe COVID-19 patients. The slightly higher average age and a higher proportion of males among patients with fungal infections than those without aligns with previous findings suggesting that both age and male sex are risk factors for severe COVID-19 outcomes. However, it should be noted that neither of these demographic variables was statistically significant in this study due to case matching, which diverges from some previous studies where age and gender were significant predictors of COVID-19 severity [49,50].

The significant difference in obesity distribution between patients with and without fungal infections is in line with numerous studies that have identified obesity as a risk factor for severe COVID-19 outcomes and a pooled risk ratio for mortality of 1.52 [51]. The enhanced susceptibility of obese patients to fungal infections could be due to a combination of factors, including impaired immune responses and an increased risk of comorbidities, as it was previously observed that a BMI over 30 is a risk factor in fungal infections [52]. In this study, a high Charlson comorbidity index (CCI > 2), indicative of multiple comorbid conditions, was significantly more prevalent in patients with fungal infections, underlining the potential role of underlying health conditions in the susceptibility to fungal co-infections [53].

The increased use of ventilators among patients with fungal infections is an important finding that aligns with previous studies suggesting that invasive mechanical ventilation may predispose patients to hospital-acquired fungal infections and significantly increase the risk of death in association with old age (OR = 2.34) and increased duration of mechanical ventilation (OR = 2.16) [54]. This is not unique to COVID-19 patients; similar observations have been reported for patients with other critical illnesses requiring mechanical ventilation. For instance, the risk of ventilator-associated pneumonia due to *Candida* spp. has been documented in patients in the intensive care unit [55]. Similarly, ventilator-associated tracheobronchitis due to *Aspergillus* spp. has been observed in critically ill patients, particularly those with chronic obstructive pulmonary disease [56]. This highlights the importance of careful monitoring and preventive measures in mechanically ventilated patients, regardless of the underlying disease condition. The significantly higher rates of ICU admission, longer ICU stays, and increased mortality among patients with fungal infections underscores the seriousness of these co-infections. Previous studies have also reported higher mortality rates among COVID-19 patients with secondary fungal infections, reaching up to 50% for infected patients according to a systematic review [57], thereby validating this study’s findings.

The findings of our study are particularly significant as they shed light on the incidence and severity of fungal co-infections among severe COVID-19 patients across six pandemic waves. Notably, we found a high occurrence of fungal infections, most notably *Candida* spp., *Aspergillus* spp., *Mucor* spp., and *Rhizopus* spp. infections, underlying the urgency for heightened awareness and surveillance of these secondary infections in severe COVID-19 cases. Arguably, the most alarming discovery in the current study was the growing trend of drug resistance among these fungal infections. Our study found that a majority of these infections exhibited resistance not only to one drug but, in some cases, two or more, a crucial finding that resonates with the larger global concern of rising antimicrobial resistance. This result signifies the urgency for more stringent antimicrobial stewardship and the potential need for innovative therapeutic approaches. The variability in the distribution of different fungal species across pandemic waves is an intriguing finding. Though this study did not find a statistically significant association, it suggests that temporal changes in the prevalence of specific fungal species may occur during a prolonged pandemic. This could be due to changes in environmental conditions, host factors, or healthcare practices and emphasizes the need for continuous surveillance during such events. Similarly, differences between the main circulating strains of the SARS-CoV-2 virus may predispose certain fungal species to co-infect or superinfect patients with COVID-19, as previously hypothesized [58]. Moreover, changes in the prevalence of specific fungal species during the six pandemic waves, although not statistically significant, point towards the possibility of varying host, environmental, or healthcare factors influencing fungal infection trends during a prolonged pandemic.

In the context of our study, the sensitivity and specificity of the multiplex PCR (mPCR) approach align with other studies that employed mPCR as a primary tool for identifying bacterial infections in severe COVID-19 patients. Our study revealed that mPCR was essential for detecting a variety of fungal pathogens, such as *Candida* spp., *Aspergillus* spp., *Mucor* spp., and *Rhizopus* spp., that occurred in the six pandemic waves. The high sensitivity and specificity of mPCR (between 95–99%), reported by other studies [59,60], are crucial in managing co-infections in patients, ensuring accurate and timely diagnoses. This is particularly significant when diagnosing co-infections in severe COVID-19 patients, where rapid identification can potentially influence the course of treatment and patient prognosis.

The study’s findings on drug resistance patterns and their association with mortality risk are particularly significant in the context of growing concerns about antimicrobial resistance globally [61,62]. The observed increase in mortality risk with increasing drug resistance aligns with findings from other studies that report a similar trend in patients with invasive *Candida* infections [63]. The issue of extensive antibiotic use, which can lead to resistance, underscores the importance of the findings in our study. We noted that a significant number of the fungal infections identified exhibited drug resistance, a fact that could potentially complicate treatment plans. This resistance was not only to one drug but, in some cases, two or more, indicating an alarming trend. Our results emphasize the importance of accurate and efficient diagnosis of co-infections through methods such as mPCR, which has proven to be a quicker and more accurate method of diagnosis [27]. Such methods can guide the selection of the most effective treatments and avoid the unnecessary use of antibiotics. It also reiterates the necessity of clinical judgment when treating COVID-19 patients with fungal co-infections, considering the complexity of potential drug–drug interactions and the increased mortality risk associated with these co-infections [64,65].

Finally, the association between specific fungal species and mortality risk offers critical insights for clinicians managing severe COVID-19 patients. The particularly high mortality risk associated with *Aspergillus* and *Mucor* infections emphasizes the importance of early detection and effective treatment of these infections [66]. This finding echoes a study published in the Journal of Fungi in 2021, which reported high mortality rates associated with *Aspergillus* and *Mucor* co-infections in COVID-19 patients.

Our results underscore the importance of rapid and precise diagnosis methods, such as mPCR, in managing co-infections in patients. We observed that mPCR was essential in detecting a variety of fungal pathogens, proving to be an effective tool in influencing treatment plans and patient prognoses. Finally, our study provides essential insights into the association between specific fungal species and mortality risk. The notably high mortality risk linked with *Aspergillus* spp. and *Mucor* infections underlines the necessity of their early detection and effective treatment. This is in harmony with previous studies, reinforcing the need for heightened vigilance and innovative therapeutic approaches for these infections in severe COVID-19 patients.

### 4.2. Study Limitations

The study presented here has a number of limitations that should be acknowledged. Firstly, the retrospective design of the study inherently carries potential biases, such as selection bias and information bias. For instance, the study only included severe COVID-19 patients, and thus, the results may not be generalized to patients with mild or moderate symptoms. Additionally, the exclusion of patients with known immunodeficiency diseases or those receiving immunosuppressive therapy may bias the results toward an underestimation of the true prevalence and diversity of fungal infections in the larger COVID-19 patient population. Furthermore, the study relied on hospital medical records, which could vary in terms of completeness and accuracy of documentation, leading to potential information bias.

Secondly, the identification of fungal infections relied solely on multiplex PCR. Whereas this is a powerful tool for detecting a range of fungi, it might not capture all possible fungal pathogens, especially those not included in the test panel, which may lead to an underestimation of the true prevalence of fungal infections. The study also did not specify the dominant SARS-CoV-2 strain during the sixth wave, which may have implications for the severity of the disease and the incidence of fungal co-infections. Finally, although the study attempted to control for potential confounders by matching patients based on age, gender, and vaccination status, other confounding factors, such as BMI and CCI, may have influenced the observed associations.

## 5. Conclusions

In conclusion, this retrospective study reveals that fungal infections, identified through multiplex PCR, are an important factor in severe COVID-19 patients, impacting both hospitalization duration and mortality rates. The presence of fungal infections was associated with a higher rate of ICU admissions, longer ICU stays, and elevated mortality rates. Among the fungal infections, *Candida* spp. was most common across all pandemic waves, but *Aspergillus* spp. and *Mucor* spp. infections were associated with a significantly increased mortality risk. It was also observed that drug resistance, especially resistance to multiple drugs, contributed to a significant increase in mortality risk among severe COVID-19 patients with fungal infections. No significant disparities were found in the distribution of patients with fungal infections across different pandemic waves, suggesting that the dominant SARS-CoV-2 variant in circulation did not significantly influence the occurrence of fungal infections. These findings underscore the importance of early detection and management of fungal infections in severe COVID-19 patients to improve clinical outcomes. Further research is warranted to optimize therapeutic strategies, considering the observed drug resistance patterns among the identified fungal infections.

## Figures and Tables

**Table 1:** General characteristics of severe COVID-19 patients with fungal infections and no fungal infections.

Variables *	Fungal Infection (*n* = 96)	No Infection (*n* = 192)	*p*-Value
Age (mean ± SD)	64.6 ± 12.1	62.0 ± 11.5	0.076
Men (*n*, %)	61 (63.5%)	107 (55.7%)	0.205
BMI obese (>29.9 kg/m^2^)	34 (35.4%)	41 (21.4%)	0.010
COVID-19 vaccinated with ≥2 doses (*n*, %)	9 (9.4%)	20 (10.4%)	0.781
Smoking (*n*, %)	42 (43.8%)	66 (34.4%)	0.121
Pulmonary disease			
Chronic bronchitis	14 (14.6%)	39 (20.3%)	0.236
COPD	18 (18.8%)	22 (11.5%)	0.092
Asthma	12 (12.5%)	20 (10.4%)	0.595
Pulmonary hypertension	3 (3.1%)	8 (4.2%)	0.663
CCI (>2)	36 (37.5%)	48 (25.0%)	0.027
Pandemic wave			0.272
1st pandemic wave	7 (7.3%)	23 (12.0%)	
2nd pandemic wave	13 (13.5%)	34 (17.7%)	
3rd pandemic wave	20 (20.8%)	30 (15.6%)	
4th pandemic wave	18 (18.8%)	29 (15.1%)	
5th pandemic wave	29 (30.2%)	46 (24.0%)	
6th pandemic wave	9 (9.4%)	30 (15.6%)	

Data reported as *n* (%) and calculated using the chi-square test and Fisher’s exact unless specified differently. * Excluding lung cancer. BMI—Body Mass Index; COPD—Chronic Obstructive Pulmonary Disease; SD—Standard Deviation; CCI—Charlson Comorbidity Index.

**Table 2:** Outcomes of COVID-19 patients with and without fungal infections.

Variables	Fungal Infection (*n* = 96)	No Infection (*n* = 192)	*p*-Value
Performed blood tests			
Conventional culture	62 (64.6%)	108 (56.3%)	0.175
Multiplex PCR	96 (100%)	192 (100%)	-
Oxygen supplementation			
AIRVO	66 (68.8%)	141 (73.4%)	0.404
CPAP	35 (36.5%)	98 (51.0%)	0.019
Ventilator	44 (45.8%)	36 (18.8%)	<0.001
Time of sampling			0.156
Within 48 h from admission	59 (61.5%)	134 (69.8%)	
After 48 h from admission	37 (38.5%)	58 (30.2%)	
Outcomes			
ICU admission	38 (39.6%)	51 (26.6%)	0.024
Days in the ICU (mean ± SD)	12.8 ± 7.2	10.5 ± 6.9	0.009
Days between symptom onset until death (mean ± SD)	13.6 ± 9.4	16.2 ± 8.0	0.014
Mortality	31 (32.3%)	23 (12.0%)	<0.001
Days until discharge (mean ± SD)	18.8 ± 9.0	15.3 ± 9.7	0.003

Data reported as *n* (%) and calculated using the chi-square test and Fisher’s exact unless specified differently; SD—Standard Deviation; PCR—Polymerase Chain Reaction; AIRVO—Noninvasive High-Flow Nasal Oxygen Therapy; CPAP—Continuous Positive Airway Pressure; ICU—Intensive Care Unit.

**Table 3:** Findings and outcomes among severe COVID-19 patients with fungal infections over six pandemic waves.

Findings	1st Wave (*n* = 7)	2nd Wave (*n* = 13)	3rd Wave (*n* = 20)	4th Wave (*n* = 18)	5th Wave (*n* = 29)	6th Wave (*n* = 9)	*p*-Value
Fungal infections							0.209
*Candida* spp.	5 (71.4%)	8 (61.5%)	7 (35.0%)	10 (55.6%)	17 (58.6%)	4 (44.4%)	
*Aspergillus* spp.	2 (28.6%)	4 (30.8%)	11 (55.0%)	6 (33.3%)	9 (31.0%)	4 (44.4%)	
*Mucor* spp.	0 (20.0%)	1 (7.7%)	1 (5.0%)	0 (0.0%)	1 (3.4%)	0 (0.0%)	
*Rhizopus* spp.	0 (20.0%)	0 (0.0%)	1 (5.0%)	2 (11.1%)	2 (6.9%)	1 (11.1%)	
Outcomes							
ICU admissions	5 (13.2%)	6 (15.8%)	8 (21.1%)	11 (28.9%)	5 (13.2%)	3 (7.9%)	0.024
Mortality	5 (16.1%)	5 (16.1%)	6 (19.4%)	10 (32.3%)	4 (12.9%)	3 (3.2%)	0.018

Data reported as *n* (%) and calculated using the chi-square test and Fisher’s exact unless specified differently; ICU—Intensive Care Unit.

**Table 4:** Antimicrobial sensitivity pattern among severe COVID-19 patients with fungal infections.

Findings	*Candida* spp. (*n* = 51)	*Aspergillus* spp. (*n* = 36)	*Mucor* spp. (*n* = 3)	*Rhizopus* spp. (*n* = 6)
0 drug resistance	10 (19.6%)	7 (19.4%)	1 (33.3%)	0 (0.0%)
1 drug resistance	18 (35.3%)	12 (33.3%)	1 (33.3%)	3 (50.0%)
2 drug resistance	20 (39.2%)	15 (41.7%)	1 (33.3%)	1 (16.7%)
≥3 drug resistance	3 (5.9%)	2 (5.6%)	0 (0.0%)	2 (33.3%)

Data reported as *n* (%) and calculated using Fisher’s exact test.

**Table 5:** Multivariate regression analysis for mortality.

Mortality Risk	OR	95% CI	*p*
By drug resistance features			
0 drug resistance	1.66	0.94–6.10	0.062
1 drug resistance	2.95	1.26–5.74	0.001
2 drug resistance	4.03	2.02–8.91	<0.001
≥3 drug resistance	6.71	1.93–10.16	<0.001
By pandemic wave			
1st pandemic wave	1.98	0.91–6.24	0.119
2nd pandemic wave	3.72	1.15–9.70	<0.001
3rd pandemic wave	1.40	1.21–4.96	0.003
4th pandemic wave	3.61	1.90–11.27	<0.001
5th pandemic wave	4.08	2.12–10.49	<0.001
6th pandemic wave	1.66	0.93–5.39	0.084
By type of fungal infection			
*Candida* spp.	1.43	0.85–2.41	0.194
*Aspergillus* spp.	4.61	1.92–13.27	<0.001
*Mucor* spp.	6.08	1.82–11.90	<0.001
*Rhizopus* spp.	5.26	0.96–6.15	0.107

OR—Odds ratio; CI—Confidence interval.

## Data Availability

Data available on request.
